# Multivariate linear regression model based on cross-entropy for estimating disorganisation in drone formations

**DOI:** 10.1038/s41598-023-39926-5

**Published:** 2023-08-07

**Authors:** Marta Gackowska, Piotr Cofta, Mścisław Śrutek, Beata Marciniak

**Affiliations:** 1 Faculty of Telecommunications, Computer Science and Electrical Engineering, Bydgoszcz University of Science and Technology, Al. prof. S. Kaliskiego 7, 85-796 Bydgoszcz, Poland; 2 Department of Informatics and New Technologies, WSB University in Gdańsk, Grunwaldzka 238A, 80-266 Gdańsk, Poland

**Keywords:** Computational science, Information technology, Computer science

## Abstract

Static formations of swarms of rotorcraft drones, used for example in disaster management, are subject to intrusions, and must bear the cost of holding the formation while avoiding collisions which leads to their increased energy consumption. While the behaviour of the intruder is unpredictable, the formation can have its parameters set to try to balance the cost of avoidance with its functionality. The novel model presented in this paper assists in the selection of parameter values. It is based on multivariate linear regression, and provides an estimate of the average disturbance caused by an intruder as a function of the values of the parameters of a formation. Cross-entropy is used as a metric for the disturbance, and the data based are generated through simulations. The model explains up to 54.4% of the variability in the value of the cross-entropy, providing results that are twice as good as the baseline estimator of the mean cross-entropy.

## Introduction

Static formations of drones are extensively used for various applications^1^, such as those involving continuous aerial observations^2–4^, the assignment of different tasks^5^, intruder monitoring^6^, air quality measurements^7,8^ or the interception of rockets and ballistic missiles^9^. The formation should be maintained even in the presence of unwanted or undesired intruders that introduce a disturbance to the formation. Anti-collision algorithms can aid the drones in avoiding an intruder by forcing them to execute collision-avoidance manoeuvres^10^ and to return to their positions in the formation.

Two of the key concerns related to drone management are the use of energy and the fulfilment of the mission. These are particularly important for battery-operated rotorcraft drones, in which the energy supply is limited. When planning a mission, it is therefore important to use a model that can anticipate the energy usage. Although such models exist^11,12^, they do not take into account the additional use of energy due to a disturbance caused by an intruder, which in some cases may be the decisive factor affecting whether the objectives of the swarm are achieved.

It should also be considered that both the parameters of the swarm and the trajectory of the intruder may affect the level of disorganisation, and thus the energy consumption and the cost. Although the managers of the swarm cannot affect the way in which the intruder approaches the swarm, they can alter parameters of the swarm itself in anticipation of the intrusion. To do this, they require guidance and information about the relationships between various parameters and the expected average disorganisation associated with the intrusion.

This paper introduces a novel model for the estimation of disorganisation, in which cross-entropy is used as a measure of disorganisation. To the best of our knowledge, it is the first model of this kind, and it offers significant improvements over existing practices that are based on experience and regulations. The model is intended to aid swarm operators, designers and managers in setting up the parameters of a drone formation to minimise the expense associated with intrusion, thus minimising energy consumption and increasing the effectiveness of the mission. A better understanding of the relationships between the parameters of the swarm, and the extent of disorganisation that can be tolerated while avoiding the intruder, may lead to better designs in terms of maintaining formations that are both effective in delivering desired outcomes and efficient in avoiding the intruder, with an acceptable loss of energy.

The novelty of this work lies in the following:

- The development of a unique disorganisation estimation model;
- The use of cross-entropy as a measure of formation disorganisation;
- A detailed examination of the dependencies affecting the degree of disorganisation.

This paper is organised as follows. The following section “Literature review” presents a literature review of stationary formation, path planning, and holding formation. “Background” section introduces background information about the anti-collision algorithm and the cross-entropy. The section “Methodology” describes the methodology used. Section “Data generation” and “Characteristics of the dataset” gives guides through the preparatory works. The development of the model is then described in section “Regression analysis”, and some results in “Results” section are presented. “Limitations to the domain” section outlines limitation to the domain. In the final “Discussion and conclusions” section, a discussion and conclusions are given.

## Literature review

This paper presents a method of estimating the disturbance caused by an intruder to a stationary swarm of drones that were running a collision-avoidance algorithm, as a disturbance of this type gives rise to additional energy expense. Thus, this work fell into two separate categories: the estimation and conservation of energy, and collision avoidance. A literature review revealed that these categories tend not to be researched together, which adds to the novelty of this paper.

The energy consumption of drones was increasingly of interest to researchers as drones become more commonplace. Papers such as^13,14^, were concerned with the use of energy by a single drone, and provided detailed models of the sub-assemblies of the drone and their energy consumption, thus allowing for the estimation of the use of energy under various flight conditions; however, they disregarded the necessity of responding to intruders and avoiding collisions. For an overview of similar models, saw also^15^.

The energy efficiency of a drone was discussed e.g. in^16^, which presented a detailed study of the relationship between energy costs and the stabilisation of the altitude and the flight speed, which were characteristic of flight in formation, but again without considering the impact of collision avoidance.

With a focus on the energy efficiency of a swarm, the work in^17^ discussed the structure of a formation that decreases the use of energy in the formation of fixed-wing drones, by exploiting the aerodynamic drag through the correct structure of a formation. The study in^18^ considered energy-efficient path planning for non-stationary formations of swarms (e.g. those used for data collection). For stationary formations, the work in^19^ presented a scheme for energy-efficient deployment. None of these works discussed the increase in energy consumption related to the intrusion or collision avoidance.

The avoidance of collisions with intruders was a widely discussed issue in relation to drones and their formations. For example, models of vehicle formation control^20^ or formation control must allowed drones to reorganised and changed their positions in order to perform specific tasks^21^, or must position robots at specific positions over time^22^. However, the additional effort (and hence energy) that the swarm must consumed in order to avoided the intruder tend to be ignored, as research in this area focused on the efficiency of the collision-avoidance algorithm.

Collision avoidance could be approached from various perspectives that combine the coordination of flight paths with the management of drones (both centralised and decentralised) at different levels of complexity. These methods could be grouped into the following classes.

1. Pre-emptive absolute path planning was a relatively simple method that involved planning flight paths for drones so that they avoided fixed obstacles as well as each other, although theywere still exposed to unpredictable and dynamic obstacles. These methods didn’t provide any way to avoid intruders, and didn’t concern with the additional burden these generate. For example, the research in^23,24^ demonstrated how the coordination of flight paths based on centralised management could be achieved using specific landmarks. Another variant of this method^25^ involved predefined flying patterns rather than paths, so that drones could resolve their location in relation to reference points.
2. Pre-emptive relative path planning offers an improvement to the static absolute planning method, in which drones were required to maintain their relative location in the swarm. This requires local cooperation between drones in a swarm, and drones therefore exchange messages about their position, speed, and identification number within given periods, to maintain a specific order^26^. Agents in a swarm could cooperate and communicate in peer-to-peer mode, and client-server communication enables them to change their configuration to the optimal one^27^. Note that enabling local cooperation was the foundation for the collision avoidance method used in this research.
3. The strategy of ‘following the leader’ offers an alternative to path planning for the whole swarm. In this case, the path was planned only for the leader of the swarm, while the remaining drones follow it. The study in^28^ showed how the topology of the formation could be maintained by keeping a constant distance and the desired angle between each drone and the swarm leader. The authors of^29^ presented a formation resembling the flocking of birds, where each agent could simultaneously act as a leader to others and as a follower to another leader. This approach allowed for some forms of collision avoidance, provided that obstacles were static and that the leader could detect them.
4. The increased autonomy of swarms calls for more autonomy in collision avoidance. If drones are aware of the locations, directions and speeds of other drones, and the possibility of obstacles, they could make autonomous decisions on how to avoid them. A well-known method was the potential field approach^30^, which was based on an analogy to charged particles in the real world, which generate a force field (electric or magnetic) that induces forces of attraction and repulsion. The study in^31^ extended the concept to a potential field with an arbitrary shape, which attracts all of the agents and prevents them from going beyond the boundaries of the field, while the repulsive force between the agents causes them to be evenly distributed so that they do not collide with each other. As the computational complexity grows, this approach could be combined with Voronoi partitions^32^ so that only the local aspects of the potential field need to be calculated. The method proposed in this research was a variant of this class of methods.
5. More sophisticated approaches combined several methods and coordination algorithms with collision avoidance mechanisms between agents in the swarm, specifically in cases of increased swarm autonomy. In^33^ a multi-cluster leader-follower approach was presented in which collision avoidance was achieved through communication between drones; each drone periodically broadcast its location and, on that basis, a safe distance between them was assured. A combination of digital maps, the locations of dynamic obstacles, and the use of artificial potential fields forming a 3D mesh allows all forms of obstacles, both stationary and mobile, to be represented uniformly by a set of points in the mesh, leading to efficient implementation of collision avoidance. The authors of^34^ presented a strategy for coordinating swarms of drones that performed a search for a target while avoiding collisions. It was based on a combination of three basic coordination mechanisms: stigmergy, flocking, and evolutionary algorithms. In^35^, an advanced flocking algorithm was presented for the coordination of drone swarms with collision avoidance, in which the parameters were optimised by an evolutionary algorithm. Artificial repulsion was used to maintain a safe distance at speed while minimising delays.

Formation holding was a special case of flying in formation, where the objective for each drone in the swarm was to remain stationary at a designated position while avoiding dynamic obstacles. This approach was essential for specialised activities such as tracking and determining the position of an intruder during a border patrol mission^26^, gathering information on traffic volumes in a smart city, where each drone shares certain information with other agents and individually decides on the next cell of the city to visit^36^, or the effective deployment of a swarm of drones, based on the theory of circular packing to act as wireless base stations^37^. An interesting case in which it was necessary to maintain a formation was a flying ad hoc network (FANET). Correct communication was very important in order to maintain a formation. An extensive and thorough systematic review of the literature related to FANETs was carried out in^38^. In this review, a FANET was defined as an ad hoc wireless network consisting of nodes, such as unmanned aerial vehicles. Although FANETs have many possibilities, there were also several limitations. Firstly, special routing techniques and protocols were required, as well as appropriate routing algorithms. The authors of^38^ indicated that algorithms inspired by biology were helpful and adequate for this purpose. Other issues related to FANETs were extensively explored in^39–42^.

The main objective of the research presented here was the development of a predictive model to link the parameters used for the formation of drones with the extent of the disruption caused by an intruder, and thus with the energy costs associated with the execution of the collision avoidance algorithm by the drones in the swarm. This model was intended to help the managers of the swarm to make decisions regarding the configuration of the swarm, and to anticipate the additional energy usage.

## Background

### Anti-collision algorithm

Regardless of any strategy applied, a drone may always be in a near-collision situation. Each drone must therefore be able to make autonomous decisions in accordance with the implemented collision avoidance algorithm^43^. However, the movement of a drone to avoid a collision may have an impact on other drones if they are close enough to be affected by its movement.

There are several existing anti-collision algorithms^44^, and the approach used in this research is a variant of a popular potential force algorithm^44^. It assumes the presence of so-called virtual security zones, referred to as $S_{1}$ and $S_{2}$. Each zone has a defined radius ($R_{1}$ and $R_{2}$, respectively), which are set as parameters in such a way as to maximise the drone’s response time and minimise the risk of a collision, especially in a dense formation. The algorithm is invoked at regular time intervals, and its outcome determines the behaviour of the drone during the next interval.

The algorithm may assume that all drones broadcast their positions, or that each drone directly observes its own vicinity. The former approach allows for larger security zones, whereas the latter is more realistic, as one cannot expect all intruders to reveal their locations. This research assumes the latter approach, and restricts the parameters accordingly.

A detailed description of the algorithm is provided in^45^. The algorithm assumes that each of the drones has a mission vector $\vec {p_{m}}$, i.e. a speed and direction that it must follow in order to execute a mission. In a stationary formation, this vector (if non-zero) is always directed towards the required location of the drone within the formation. Furthermore, the drone knows its position $x_{j}$ and observes its own vicinity. When there are no drones in its neighbourhood, the mission vector is executed. If there is any obstacle within the zone $S_{1}$ of the drone, it executes only the escape vector $\vec {p_{u}}$ (Eq. 1). When there is an obstacle within the zone $S_{2}$ of the drone, the vector $\vec {p_{z}}$ is used (Eq. 2), so that both the mission and the escape are carried out. Otherwise, the drone follows the mission vector $\vec {p_{m}}$.

1 $$\begin{aligned} \vec {p_{u}} = \frac{\vec {p_{l}}}{|\vec {p_{l}}|} (R - |\vec {p_{l}}|)^{q}, \end{aligned}$$

where $$\vec {p_{l}} = (\vec {x_{1},y_{1}})$$

2 $$\begin{aligned} \vec {p_{z}} = \vec {p_{m}} + \tau * \vec {p_{u}} \end{aligned}$$

In contrast to the original version of the anti-collision algorithm^43^, a modification is introduced here so that if any drone is found in the inner security zone, the escape vector is turned slightly anticlockwise. This results in higher efficiency of the collision avoidance response, without altering its nature. In addition to $R_{1}$ and $R_{2}$, this algorithm relies on two parameters, $\tau$ and q. The parameter $\tau$ is a linear element, whereas the parameter q is a nonlinear one. It can be assumed that they define the degree of “nervousness” of the drone: if both parameters q and t are reduced, then the drone becomes tolerant, and has a sluggish reaction to the behaviour of the environment; on the other hand, when these parameters are increased, the drone becomes excessively “nervous” and reacts too intensely.

### Cross-entropy

In this research, we use cross-entropy as a comparative metric of the disturbance within the swarm. The usefulness of this metric was previously established in^45^, including for situations caused by an intruder to a stationary formation of drones. The cross-entropy represents the difference between the expected and the actual distribution, in this case the distribution of drones in space. The general expression in Eq. 3 is used to calculate the cross-entropy:

3 $$\begin{aligned} H(p,q) = - \sum \limits _{x\in X} p(x)*log(q(x)) \end{aligned}$$

where p(x) represents the expected distribution and q(x) the actual distribution. Consequently, the cross-entropy of a stationary formation relates to the distance between the actual and the expected positions of the drones.

A relationship is assumed between the level of cross-entropy and the additional expenses that the swarm must bear in the form of additional energy consumption, communication overhead etc. Generally It has been demonstrated that the higher the cross-entropy, the greater and more costly the changes to the formation of the drone swarm caused by the intruder and the operation of the collision avoidance algorithm. Thus, cross-entropy can be used as a measure of the cost of holding the formation in place.

The cross-entropy is calculated at each step of the simulation, for all the drones in the formation. The reported cross-entropy is the sum of these individual cross-entropies. For a situation where there is no intruder, the expected cross-entropy is zero, as the positions of the drones do not change. Note that the cross-entropy is a comparative metric rather than an absolute one. Although the relationship between the actual additional expense and the value of the cross-entropy has not been yet established, we know that the higher the cross-entropy, the higher the additional expense.

## Methodology

The objective of this research was to develop a model, based on linear regression, that allowed for the prediction of the average disturbance caused by an intruder, on the basis of the parameters selected for a swarm.

**Figure 1:**
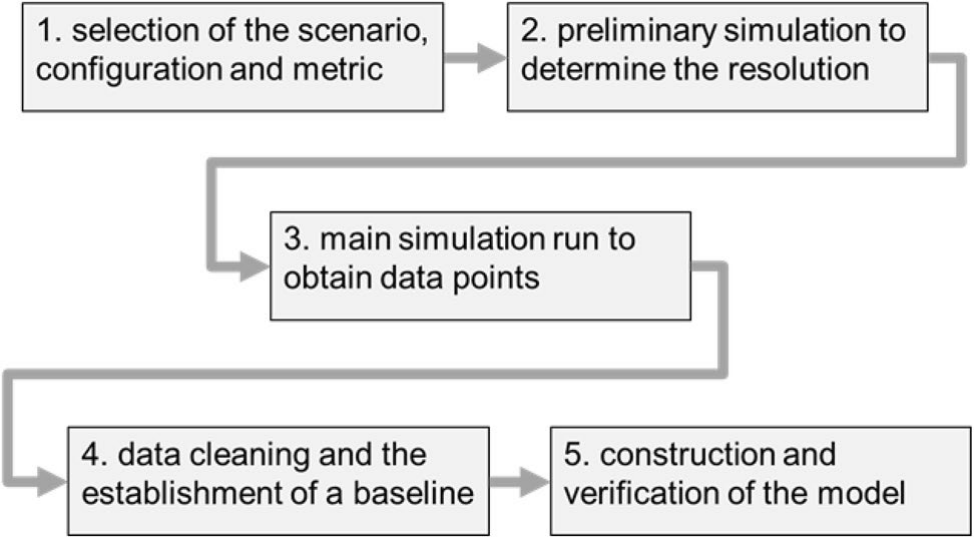
The methodology of the research.

The research followed the five-stage process, shown in Fig. 1. In (1), on the basis of previous research^45^, a single corner scenario was selected. It determined the configuration as well, including the range of parameters. Cross-entropy was chosen as a metric of disturbance, as this closely reflected the additional overhead imposed on the swarm to overcome the disturbance. In (2), an initial investigation of the behaviour of the swarm was conducted with the help of a simulator^45^ to determine the appropriate resolution of sampling and to validate the expected near-chaotic nature of the formation.

In (3), the simulator was used to obtain more than 3000 data points, which were used to create a dataset. Each data point required approximately 250,000 simulations, as the parameters of the intruder was unpredictable. Data cleaning (4) followed, and data points that did not minimum quality requirements were removed. The baseline of mean and median values was established. Finally, in (5), the model was constructed using several multivariate regression algorithms, using the dataset as a ground truth. Results were assessed using previously selected metrics of $R^2$, MSE and MAE.

## Data generation

### Simulator

The dataset used to develop the model was created with the help of a 2D (i.e. single-layered) simulator^46^, which was developed by the current authors^45^. This allowed for the simulation of several scenarios, such as the passage of an intruder through a formation, manoeuvres involving several formations, the passage of single drones, etc. Each group of drones may (or may not) execute a collision-avoidance algorithm^43^, potentially with different sets of parameters. The simulator provided a record and a visualisation of the behaviour, and calculated the cross-entropy metric for selected groups of drones. The simulator was deterministic: two runs of the simulation with identical parameters led to the same behaviour and the same cross-entropy.

### Scenario

A single scenario was considered throughout the whole study, and was selected on the basis of previous research as a particularly complicated one. The scenario involved a 2D swarm of drones, that maintained a rectangular formation. The intruder was assumed to be running no anti-collision algorithm, while all of the drones within the formation were running an anti-collision algorithm. The intruder always approached the formation from its lower left corner. The angle of approach was varied from 0$^{\circ }$ to 90$^{\circ }$, where 0$^{\circ }$ implies approach along the x-axis, and 90$^{\circ }$ along the y-axis. The intruder’s point of entry was offset relative to the location of the lower left drone of the formation by no more than half of the distance between drones, either horizontally or vertically.

**Figure 2:**
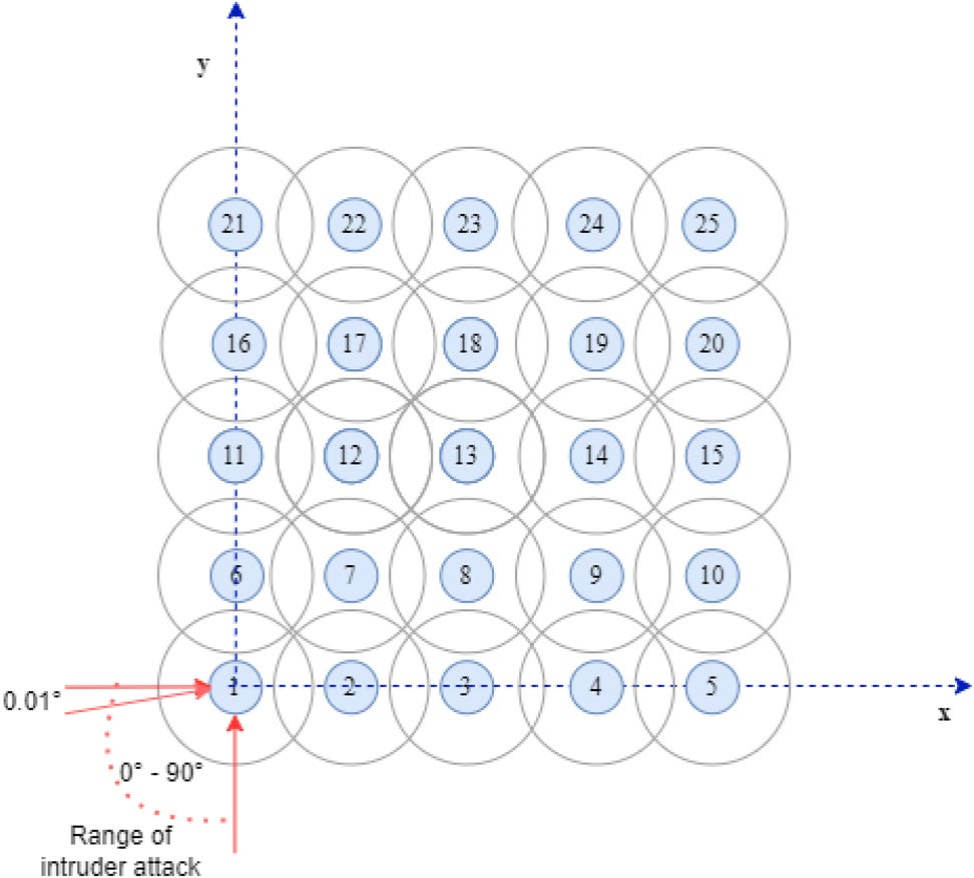
Diagram showing the angle of approach.

In order to illustrate the concept of the angle of approach, an example is shown in Fig. 2. The intruder always flew straight towards the drone (so that the offset was zero), albeit at different angles. The formation consisted of 25 drones spaced at intervals of 30 m, starting at the position (30, 30).

**Figure 3:**
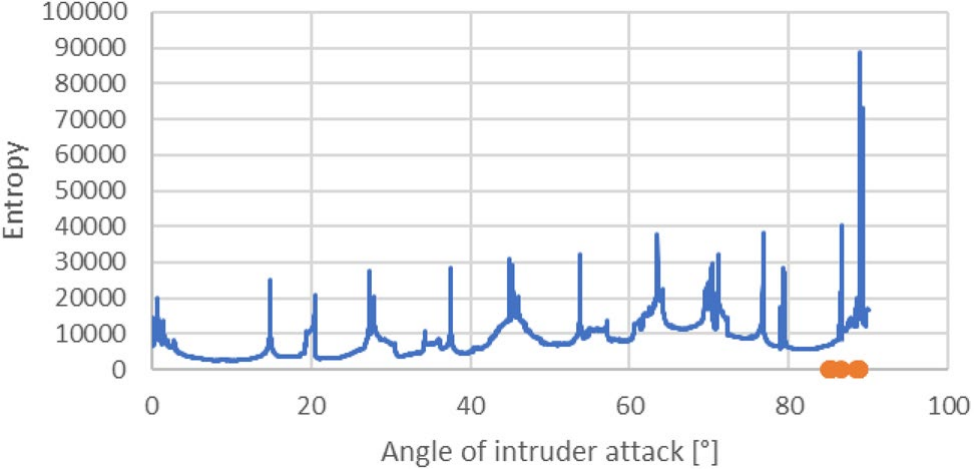
Relationship between angle of approach and cross-entropy.

It is worth considering the impact that changes in the angle of approach may have on the cross-entropy. Figure 3 shows the cross-entropy calculated for each 0.01$^{\circ }$ change in the intruder’s angle of approach. The parameters for the implemented collision avoidance algorithm were: $R_{1}$ = 15 m, $R_{2}$ = 20 m, $\tau$ = 1, q = 1.

The collisions occurring due to the flight of the intruder drone are marked with orange dots, and cross-entropy is not shown for these angles. However, the occurrence of a collision is associated with a significant increase in the disorganisation of the formation for neighbouring angles to those at which the cross-entropy reaches the highest values. Moreover, Fig. 3 shows that situations with high cross-entropy are very close to situations with relatively low values, meaning that even the slightest change in the angle may result in a significant change in cross-entropy.

**Figure 4:**
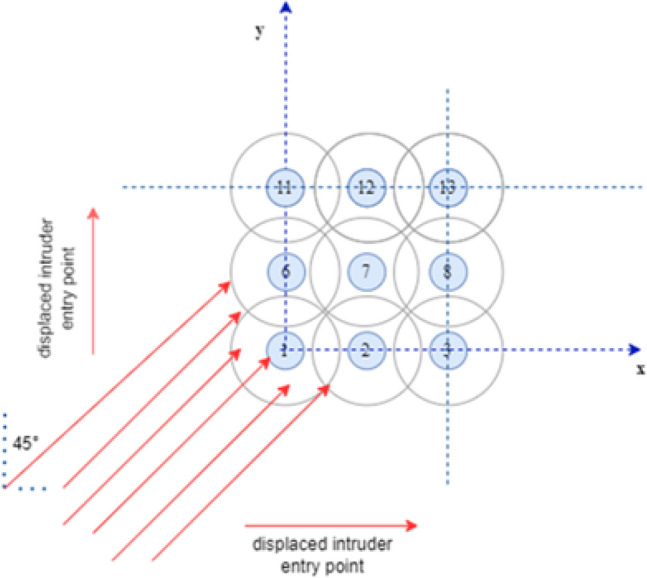
Diagram showing the point of entry (only a fragment of the swarm is shown).

A similar analysis could be carried out by observing changes in the cross-entropy resulting from slightly altering the offset of the point of entry of the intruder, as shown in Fig. 4. In this case, the intruder drone approached the formation at a constant angle of 45$^{\circ }$, and the range of entry points extended to half the spacing in both directions.

**Figure 5:**
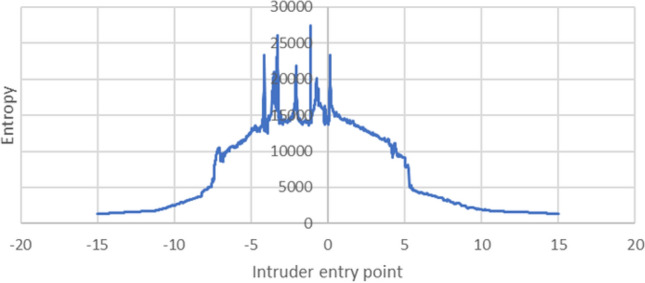
Relationship between entry point and cross-entropy.

Figure 5 shows the relationship between the point of entry and the cross-entropy described in terms of the offset, as shown in Fig. 4. Again, cases in which the cross-entropy reaches high values are close to those with low values, indicating that even a small change in the offset of the entry point may result in a significant change in the cross-entropy.

### Parameter domain

The applicability of the model is limited to its domain, which is defined by the ranges of parameters shown in Table 1. These ranges were determined on the basis of prior research, with the aim of concentrating on problematic situations while preserving the rules of flight safety. In other words, these ranges reflect what the managers of the swarm may consider typical and appropriate in the course of their work.

**Table 1:** Parameters and their ranges.

Parameter	Min value	Max value
Formation	Rectangular	
Size (drones in any row/column)	2	9
Spacing (distance between drones) (m)	25	50
$R_{1}$ [outer radius, collision-avoidance algorithm (m)]	13	$R_{2}$
$R_{2}$ (inner radius) (m)	20	Spacing
$\tau$ (reaction)	0.4	1.4
q (acceleration)	0.4	1.4

Note, that for parameters $R_{1}$ and $R_{2}$, the upper limit in each case is determined by the value of a different parameter. This limitation reflects the way the collision-avoidance algorithm works. Values of $R_{1}$ and $R_{2}$ that exceed these limits lead directly to undesirable behaviours of the swarm, such as collisions and vibrations, as described later.

In practice, managers often enforce an additional safety gap between these parameters, to take account of the possible imprecision of the measurement, inertia, or the impact of the wind. Although the model itself does not include this gap this paper presents additional research analysing the impact of such a gap on the quality of the model and estimations.

### Sampling interval

Each single data point represents the result of a large number of simulations. For a given combination of parameters, different angles of approach and points of entry must be considered. Both the angle and the point are continuous variables that can take any value from a given range. In order to achieve comparable results for all data points, and to limit the number of simulations, it was necessary to introduce a sampling interval to guide the selection of angles and points for the simulations.

Due to the near-chaotic relationship between these parameters and the cross-entropy, as illustrated in Figs. 3 and 5, determining such an interval required additional research. A data point with an average combination of parameters was chosen, and its cross-entropy was then calculated at intervals of 0.001$^{\circ }$ and 0.001 m for the angle and the entry point, respectively, in subsequent simulations. Although these intervals were too small for practical purposes, as they were below the precision of instruments used to determine the angle and the location, they will serve as a good reference should such precision become attainable.

The average cross-entropy obtained from these simulations was taken as a reference value. The set of simulations was then run again for the same parameters, with increasingly large intervals, and the results were compared. The metric was the measurement error (MAE), which was calculated as the difference between the reference cross-entropy and the cross-entropy calculated using this increment. For reasons of practicality, a sampling interval of 0.05 or greater was desirable, as the larger the interval, the faster the generation of the data point.

**Figure 6:**
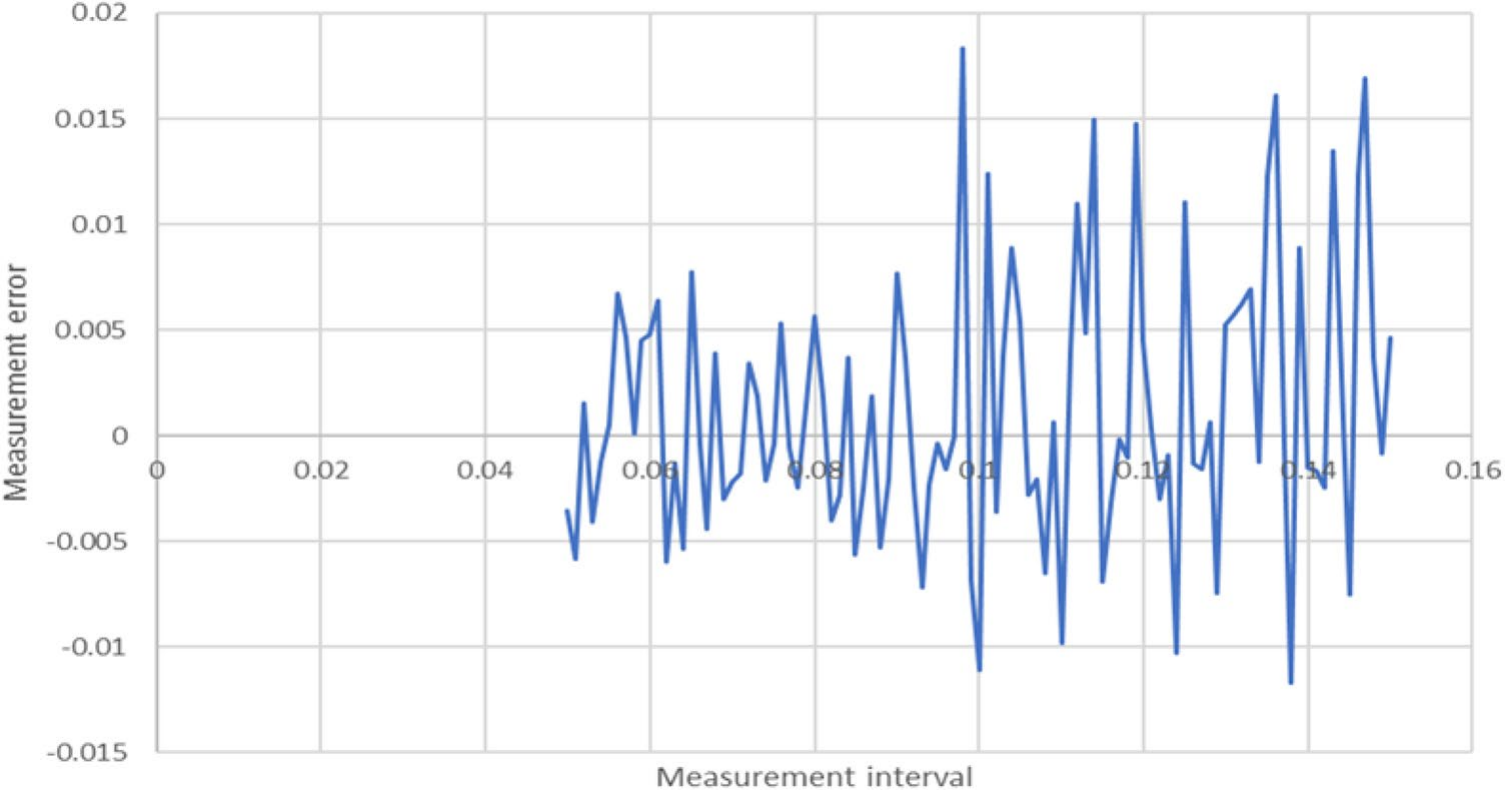
Relationship between the interval and measurement error for the angle of approach.

**Figure 7:**
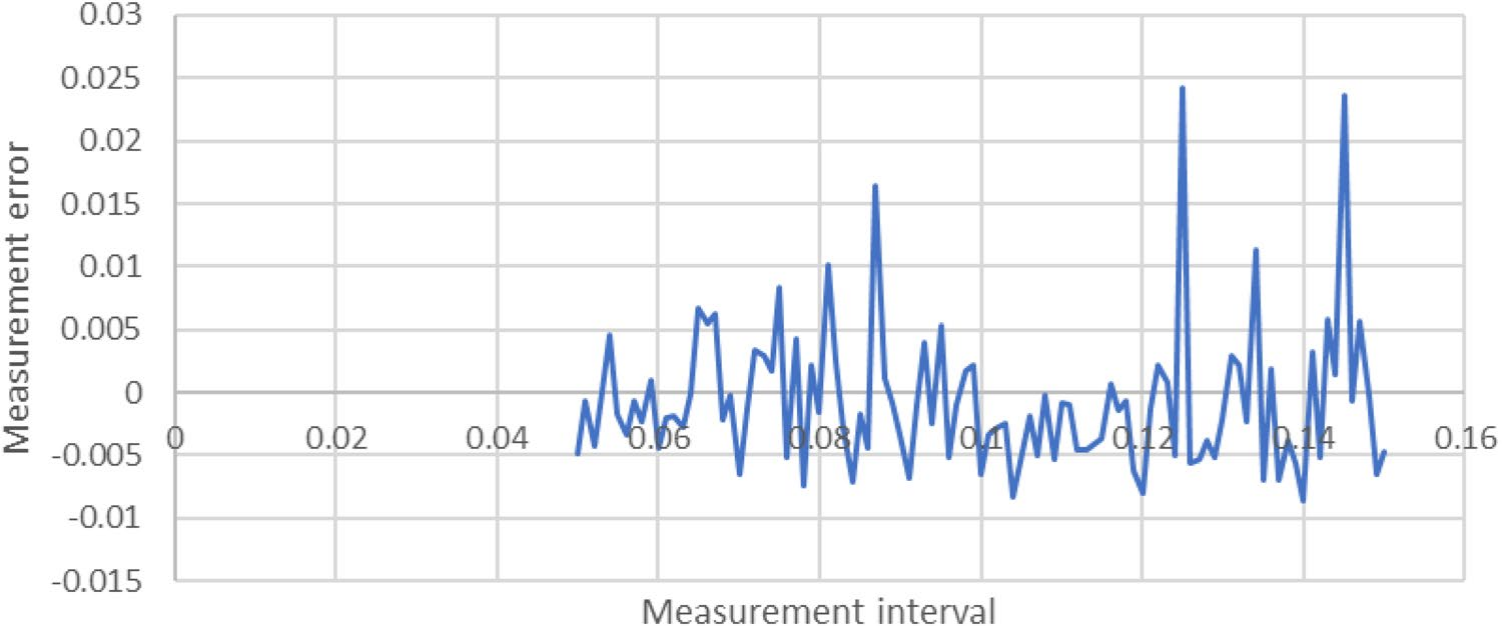
Relationship between the interval and the measurement error for an entry point.

The results are shown in Figs. 6 and 7 for the angle and the entry point, respectively. As expected, no clear relationship was observed between the interval and the level of error, but there were clear boundaries on the maximum value of such an error. The error associated with the increase of the sampling interval was within $\pm \, 2\%$ for the angle, and between + 2.5% and 1% for the entry point, across a wide range of intervals. Eventually, sampling at intervals of 0.1 was chosen for both the angle and the entry point, with the expectation that the introduced error would be less than 5%.

### Data points

The outcome of these simulations was a set of data points. Each single data point was the result of 270,000–450,000 simulations, and was stored as a record in a CSV file with columns as shown in Table 2.

**Table 2:** Data points with description.

Id	Name	Description	Comments
0	Formation type	Constant ‘RECTANGLE’	RFU
1	Formation index	Constant 0	RFU
2	Size	Size of the swarm, number of drones in each row/column	
3	Spacing	Distance between adjacent drones (m)	
4	$R_{1}$	Inner radius of the collision-avoidance algorithm (m)	
5	$R_{2}$	Outer radius of the collision-avoidance algorithm (m)	
6	$\tau$	Parameter of the collision avoidance algorithm	
7	q	Parameter of the collision avoidance algorithm	
8	Collision	Estimation of the probability of collision for a given set of parameters	Fields 8 to 11 sum to 1.0
9	Oscillation	Estimation of the probability of oscillation for a given set of parameters	
10	Vibration	Estimation of the probability of vibration for a given set of parameters	
11	Passage	Estimation of the probability of passage for a given set of parameters	
12	Cross-entropy	Average cross-entropy for all passages within this data point	

## Characteristics of the dataset

The resulting dataset contained 3720 unique data points. A brief analysis revealed that datasets with less than 1000 data points did not deliver consistent results, so the whole dataset was used.

### Outliers and data cleaning

The identification and removal of outliers is important, as their existence affects the outcome of the analysis. The literature describes three different approaches to the identification and removal of outliers:

- If there are known deficiencies in the data gathering process, data points affected by those deficiencies are clear candidates for removal;
- If there are certain physical or practical impossibilities that restrict the correct values, those restrictions may indicate outliers;
- Finally, if little is known about the data collection process or the phenomenon itself, a statistical analysis can be used to determine outliers; however, this approach may remove data points that are of value, so it should be used with care.

The process used for the removal of outliers in this research drew on the first two of these approaches. Although the dataset was generated by a simulator, some outliers were still possible due to the inherent limitations of the averaging process as well as the simulation itself. Two forms of outliers were identified, and were dealt with as follows.

- There were situations where, for a given set of parameters, every attempt of the intruder to pass through the swarm ended in collision or oscillation, meaning that the number of successful passages was zero. In this case, it was not possible to calculate the average cross-entropy for the passage, and the simulator in those cases reported a cross-entropy of zero. As the objective of this research was to establish a model to estimate the cross-entropy for a successful process, these data points were removed from the dataset.
- There were also situations where the fraction of successful traversals was so low that the average value obtained for the cross-entropy from the simulator could be highly uncertain. The authors resolved to remove datasets where the fraction of successful passages was lower than 0.01 of the total number of attempted passages, which resulted quite often in the removal of data points with very high cross-entropy. In a later section of this paper, the rationale for this decision and its impact on the dataset are discussed.

All in all, only about 4% of the data points were classed as outliers and were removed.

### Basic parameters

When the outliers had been removed, the dataset had basic statistical properties as shown in Table 3.

**Table 3:** Basic parameters of the dataset.

Parameter	Value
Count	3720
Median cross-entropy	13,226.03
Mean cross-entropy	22,191.13

**Figure 8:**
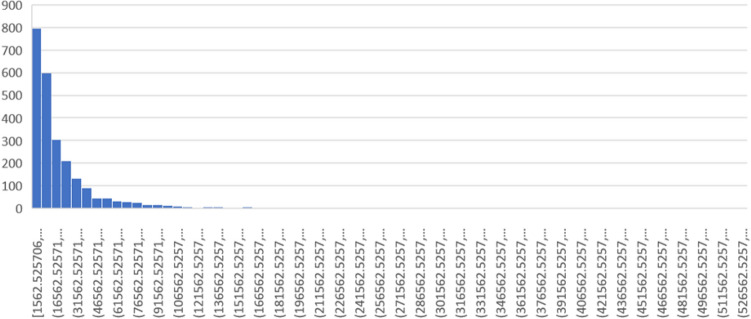
Histogram of cross-entropy.

A histogram of the distribution of cross-entropy values is shown in Fig. 8. It is apparent that the distribution is heavily slanted towards smaller values, but has a long tail. This distribution could be approximated using a power function $y=3322.9* x^{-2.393}$ with $R^2=0.7347$.

**Figure 9:**
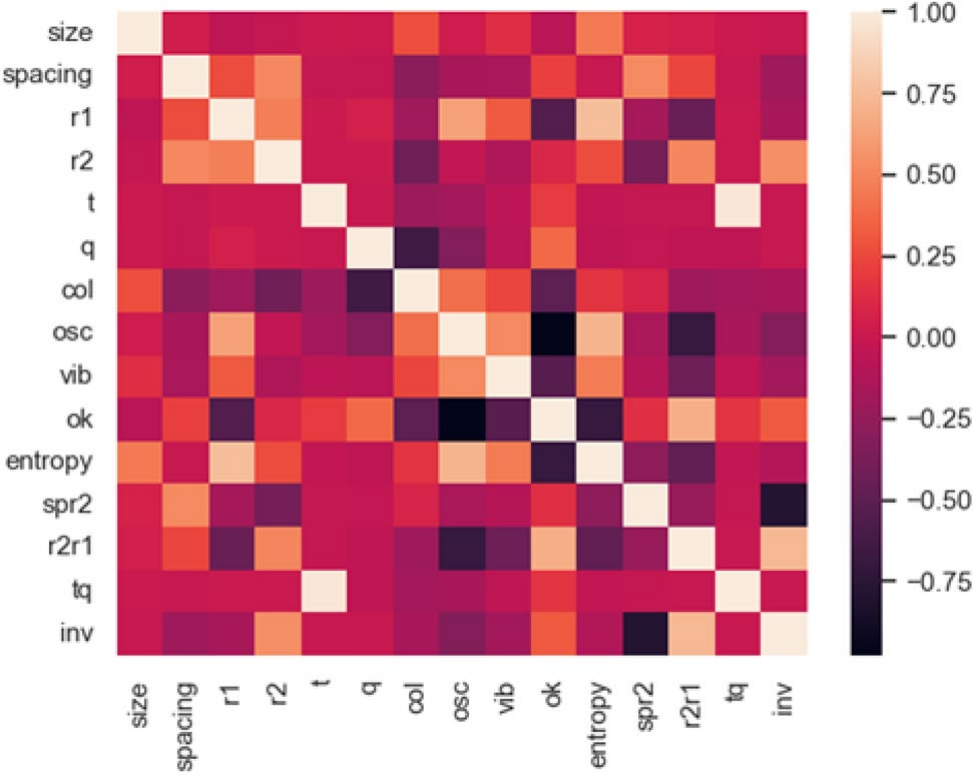
Correlation heatmap, including derived values.

The correlation heatmap in Fig. 9 represents the expected relationships. The values for the derived parameters (described later) are correlated with the values of the components. A negative correlation was also noted between the probability of oscillation and the probability of a passage, which was as expected, since these probabilities and the probability of vibrations sum to one. For the cross-entropy, there were positive correlations with $R_{1}$ (0.64) and oscillations (0.66), and a negative correlation with the passage (- 0.64). It can therefore be expected that large values of cross-entropy will be correlated with situations that border on oscillations, where passage is not possible or is very unlikely. These situations may be driven by the size of $R_{1}$.

### Nonlinearity

The histogram indicates nonlinearity in the relationships between the parameters and the cross-entropy. We observed the behaviour of the system under various conditions, and concluded that the swarm was likely to be at the edge of chaos, as a very small change in initial settings (i.e. in the parameters) may lead to significant changes in the behaviour of the system, thus explaining the variability in cross-entropy. Since the system is algorithmic with no random (or pseudo-random) component, and is not significantly affected by rounding errors, this system can be classified as a nonlinear deterministic system.

As described in the literature, a system is on the verge of chaos when a small change in the initial conditions causes large but limited differences in the results^47^. For example, in a study of multi-agent traffic evolution^48^ the model was deliberately put into a state on the brink of chaos, due to an increase in system efficiency and a reduction in the agents’ travel time.

The observable behaviour of such systems can usually be described by a set of nonlinear equations, and can be approximated by a set of linear ones if linearisation is applied and/or if the model is limited to a specific subset of initial states. Consequently, we did not expect linear regression to achieve a particularly good estimation. However, linear regression was expected to give an improvement over the baseline of the naïve mean or median estimator, and to serve later as a reference when more advanced methods of estimating the observable output are developed.

### Collisions, oscillations and vibrations

There are two types of behaviour that prevent the grid from correctly calculating the cross-entropy: collisions and oscillations. As this research focuses on cross-entropy, both of these situations are excluded from consideration here.

- Collision is defined as the situation where two drones (most likely an intruder and a drone from the formation) reach such a proximity that neither are able to continue their operation. From the perspective of a model, collisions do not have an cross-entropy associated with them, as the occurrence of a collision negates the validity of a set of parameters. Note that while care has been taken to choose parameter values that do not facilitate collisions, these can still happen.
- Oscillations emerge from behaviour where, for example, a drone moves significantly away from its initial position due to being knocked out of the formation by an intruder drone. Subsequently, it is unable to return to its position, as it is blocked by the other drones in the swarm.
- Finally, vibrations are situations where the drones in the formation are unable to return to their exact positions, for instance because they are continuously affected by nearby drones, with no chance of the situation eventually stabilising.

A situation involving a collision, oscillation or vibration relates to a single simulation, and does not negate the value of an overall data point, which is based on the average of thousands of simulations. In the dataset used in this research, each data indicates the probability of occurrence of these unwanted phenomena among all its simulations. Each of these three phenomena invalidates the value of the cross-entropy for a given simulation, which is excluded from the average.

## Regression analysis

The objective of this study was to attempt to use linear regression as an estimator of the average cross-entropy of a swarm. In view of the number of possible linear regression algorithms, and the numbers of parameters they require, the following four multivariate algorithms were considered. Simple linear regression: This algorithm was selected to serve as a baseline. It is the simplest option, and minimises the ordinary least squares function. This algorithm is known to perform well for data with a normal or near-normal distribution, which is not the case here. Further, the number of data points needs to be fairly large. We therefore did not expect that this algorithm would perform particularly well, but that it would establish expectations for the behaviour of other algorithms.

*Ridge regression* This algorithm is suitable for handling situations where independent variables are correlated. The heat map indicated that there were no strong correlations between the parameters themselves, although the derived variables were correlated with the parameters. Consequently, there was an expectation that this algorithm would outperform simple regression, as these variables were included. The implementation of this regression allowed for the choice of solvers. Preliminary studies indicated that the singular value decomposition (SVD) solver slightly outperformed the others.

*Huber regression* The inclusion of this algorithm was driven by its well-known ability to handle situations with outliers, where the term ‘outlier’ does not refer to a suspicious data point but simply one that lies far from the centre of the distribution. As the dataset contained data points of this kind, it was expected that this algorithm would outperform the others. Preliminary research was conducted to find the optimum set of parameters, and values of epsilon = 2.1 and alpha = 0.01 were chosen, with the maximum number of iterations limited to 1000.

Theil-Sen regression: This choice was based on the fact that this algorithm is believed to be less sensitive to outliers, similarly to the Huber algorithm. Furthermore, Theil-Sen is median-based, and in this case, the median was expected to give a better estimate considering the distribution of the dataset.

### Baseline

In order to establish whether it was possible to improve on a given estimator, it was important to establish a baseline to establish expectations. For systems that are close to chaos, estimators can be hard to establish, and as a baseline, the simplest possible estimator (i.e. the mean and median cross-entropy, calculated across all combinations of parameters) was therefore selected as a baseline. Values were not normalised. Three error estimators were used to assess the outcome: $R^{2}$, mean absolute error (MAE) and mean squared error (MSE). For reference, the values of the mean, median and respective error estimators are provided in Table 4 below.

**Table 4:** Naive estimators and their errors.

Estimator	$R^{2}$	MAE	MSE
Mean	0.000	16,877.94	813,783,995.93
Median	$-$ 0.099	14,943.95	894,157,017.33

It is interesting to note that none of these estimators is clearly the best. The mean gives slightly better results for $R^{2}$ and MSE, while the median is better when the MAE is considered. For such a skewed distribution, it could generally be expected that the median would provide better results.

Note that for a truly chaotic system, even this kind of estimator offers an advantage, as it assumes that the behaviour of the system, despite being perceived as chaotic, is statistically predictable. Finding a better estimator than this one may mean that there is actually less chaos than perceived by the observer.

### Derived variables

As indicated earlier in this paper, the application domain was intentionally constrained to specific ranges of values for six parameters: size, spacing, outer radius ($R_{2}$), inner radius ($R_{1}$), and two parameters that control the response to the impeding collision: $\tau$ and q. However, an understanding of how the swarm operates led to the introduction of three derived variables, in the expectation that they would better capture the relationships present in the system, specifically those that are believed to be nonlinear. Those are as follows:

- $D_{1}$: spacing–$R_{2}$. This variable represents the range over which drones are not within the sensing range of their neighbours, so that any movement of one drone within this range does not affect other drones. This arose from the early observations of the cascading effect, where the movement of one drone rapidly propagates through the swarm and may lead to a significant increase in cross-entropy.
- $D_{2}:R_{2} - R_{1}$ This variable represents the tolerance of a drone in terms of switching from a strategy of avoidance to pure escape from other drones. Drones with high tolerance tend to respond more slowly and with more precision to incoming drones, while a low tolerance means that their responses overshoot.
- $D_{3}: \tau ^{q}$ This variable was introduced to represent the apparent nonlinearity in the model, as the actual speed of response of a drone depends linearly on the value of $D_{3}$, rather than on $\tau$ and q.

We note that experiments were conducted with and without these derived values, to explore whether they actually improved the operation of the estimator.

### Linearisation

Some forms of nonlinearity can be corrected by linearisation. In this research, simple linearisation of the individual independent variables was attempted, as the development of a completely nonlinear model was outside the scope of the study. A visual inspection of the scatterplots did not reveal obvious candidates for linearisation, although there were strong candidates where the averaged cross-entropy was compared to the values of parameters. Finally, the transformations shown in Table 5 were applied.

**Table 5:** Linearisation.

Parameter	Transformation	$R^2$
Size	y = 122.75x3 − 2601.3x2 + 19506x − 22590	0.9932
Spacing	n/a	
$R_{1}$	n/a	
$R_{2}$	y = − 18.791x2 + 2403.7x − 2922	0.6627
$\tau$	n/a	
q	n/a	
$D_{1}$: spacing – $R_{2}$	n/a	
$D_{2}$: $R_{2}$ – $R_{1}$	y = 99.413x2 − 3764.2x + 43333	0.8476
$D_{3}$: $\tau ^q$	n/a	

### Metrics

Three error estimators were used to assess the outcome: $R^2$, MAE and MSE. All three estimators were used only for comparison.

- $R^2$ was chosen as a standard estimator of the extent of the variability that was explained by the model.
- MAE was chosen due to the presence of large values or outliers, in the expectation that it would provide a more realistic estimation.
- MSE was selected to provide a way to compare our results with those achieved by other methods, based on the understanding that the existence of outliers may significantly alter the value of this estimator.

## Results

Our results are summarised in Table 6, which also contains the baseline results presented above in Table 4. For each algorithm, four variants were tested to see whether any improvement was achieved by the inclusion of the derived variables and linearisation. Values are rounded to two decimal places, or three places for $R^2$. The best results for each metric are shown in bold.

**Table 6:** Summary of results.

Estimator	Derived	Linearised	Metrics		
			R2	MAE	MSE
Mean			0.000	16,877.94	813,783,995.93
Median			− 0.099	14,943.95	894,157,017.33
Linear			0.502	10,225.37	405,191,942.21
		x	0.496	10,302.49	410,246,564.24
	x		0.527	9876.90	384,927,025.85
	x	x	**0**.**544**	9761.51	370,809,271.72
Ridge			0.502	10,202.09	405,116,735.57
		x	0.496	10,278.44	410,172,210.19
	x		0.527	9861.38	384,865,270.86
	x	x	0.544	9728.00	**370,718,973.68**
Huber			0.470	9096.48	431,711,688.36
		x	0.441	9532.31	454,474,848.82
	x		0.491	8545.96	414,336,654.15
	x	x	0.484	8748.43	420,299,390.05
Theil-Sen			0.445	8919.57	451,677,447.79
		x	0.434	9005.75	460,584,362.45
	x		0.479	8386.25	424,053,353.48
	x	x	0.486	**8277.37**	418,294,361.24

Based on the naive estimators (the mean and the median), we see that all algorithms were improved by about 50% across all of the error metrics. This indicates that there is an untapped linearity in the otherwise chaotic behaviour of the swarm, which can be captured by linear regression. If no better estimation is required, these algorithms can establish reasonable expectations for the cross-entropy of the swarm.

There was no clear winner among the linearisation algorithms; regardless of the metric used, the difference between the best and worst performers did not exceed 20%. Linear regression with derived variables and linearisation achieved the best value of $R^2$, while Theil-Sen regression with derived variables and linearisation gave the best value of the MSE, and the Huber algorithm with derived variables and linearisation was the best when measured based on the MAE.

The impact of additional variables and linearisation did not exceed 5%, meaning that it was within the level of the ‘measurement noise’, which was established earlier to be 5%. It can therefore be concluded either that this problem cannot be easily improved by these methods, or that further research should be conducted. In view of the nonlinear nature of the problem, the meagre gain of 5% is likely to reaffirm the earlier observation that an entirely nonlinear regression method may be a better avenue for exploration.

## Limitations to the domain

The ranges of the parameters that were considered in this research were intentionally left fairly wide, leading to situations that were likely to give increased errors. Some of these problematic situations were addressed by the introduction of derived parameters and by the elimination of outliers. As no regression algorithm performed particularly well, it is worth exploring the possibility of further restricting the ranges of the parameters, in an attempt to allow the model to perform better, although for a more constrained set of situations. In light of this, the potential impacts of the following two choices were worthy of consideration.

The ‘safety gap’ left between the spacing and $R_{2}$, as well as the difference between $R_{2}$ and $R_{1}$, have already been discussed in conjunction with the derived parameters $D_{1}$ and $D_{2}$. From observations of the swarm, it was clear that the nonlinearity was mostly associated with small sizes of this gap. In such situations, the swarm is very likely to undergo collisions or oscillations, and significant problems with any forms of a passage of an intruder are likely to arise. As managers and operators of the swarm are unlikely to use such small gaps in practice, the introduction of such a gap may be warranted.

The removal of outliers, discussed previously in this paper, set the minimum acceptable fraction of passes to above 0.01. Although this means at least 2500 passes, this number of samples may not be sufficient to establish a reliable average. Increasing the required fraction may decrease the number of data points, but may also make them more reliable.

### Safety gap

It has been already mentioned that a safety gap was left between certain parameters to minimise the occurrence of undesired effects. The existence of this gap alters the probability distribution of parameters in the sample, and eliminates infrequently occurring data points with very high values of cross-entropy.

It may also therefore have an impact on the results of regression, at the expense of limiting the domain. Additional research was conducted to explore whether this impact was significant. The same dataset was selectively cleaned of data points that did not meet the criteria for the gap, which was varied between zero and 7 m. Only the $R^2$ error estimator was used, and only for simple linear regression, as our previous results indicated the sufficiency of this approach.

**Figure 10:**
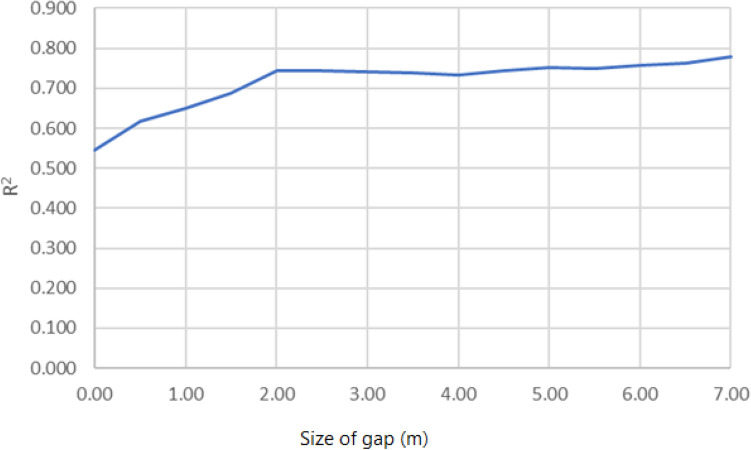
Relationship between the width of the safety gap and $R^2$.

Figure 10 illustrates the relationship between the width of the gap and the value of $R^{2}$. It is clear that the introduction of a relatively modest gap of 2 m has a significant impact on the model, increasing $R^{2}$ from about 0.55 to about 0.75. Further increases in the gap have no apparent impact. It can be expected that the impact on the other estimators will be equally positive. While this gap is sometimes used in practice, it is not yet commonly applied. We therefore decided not to introduce the requirements for the gap directly into the model, but rather to present them here as a possible avenue for improving the quality of the model.

### Fraction of simulations excluded

Each data point was constructed based on a large number of individual simulations. However, simulation results that included collisions, oscillations or vibrations were excluded, as the values of the cross-entropy for those data points were less certain. It was possible to remove from the datasets the data points with higher uncertainty, measured here as the excluded fraction of simulations.

For all the results presented earlier in the paper, the minimum acceptable fraction of passes was set within the model to 0.01. That is, data points that for which the simulations did not indicate passes in at least 1% of cases were removed from the dataset.

A separate experiment was conducted in which simple linear regression was applied to a dataset from which these data points had been removed, where the ‘excluded fraction’ was varied between zero (i.e. no exclusions, regardless of the uncertainty) and 10% (i.e. it was necessary for at least 90% of the simulations of the data point to end in the passage of the intruder). $R^{2}$ was used as an indicator of the quality of the regression.

**Figure 11:**
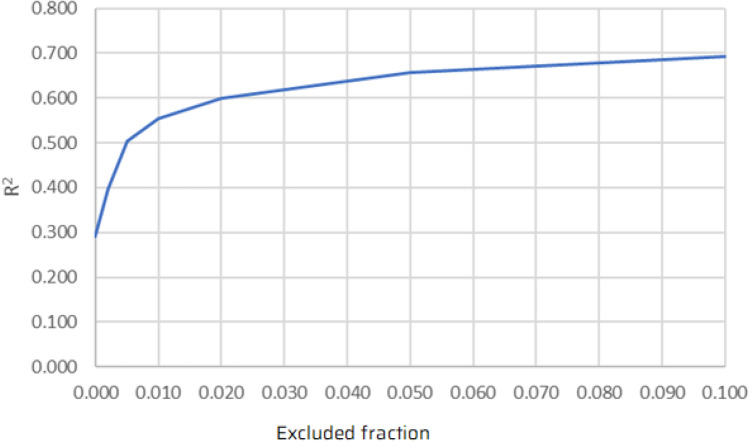
Relationship between the excluded fraction and $R^2$.

Figure 11 shows the relationship between the excluded fraction and $R^{2}$. It can be seen that the exclusion of data points with higher uncertainty had a positive impact on the quality of regression. For example if 10% exclusions are allowed, linear regression can deliver $R^{2}$ at the level of almost 0.7.

Both of the methods described above lead to an improvement in the predictability of the model at the expense of the domain of applicability. There was a very high correlation of about 0.97 between the results achieved by the two methods, specifically for small values of the gap and the excluded fraction.

## Discussion and conclusions

The model presented in this paper was designed to aid a manager of a swarm formation of rotorcraft drones in correctly setting parameters to minimise the overhead caused by the passage of the intruder. The model usues multivariate linear regression, and is based on data from simulation.

The model delivered a two-fold improvement in the prediction of the cross-entropy (as measured by the MAE), and about 50% of the variability could be explained by it.

To the best of our knowledge, it is the first model of its kind, and delivers significant improvements over existing methods that are based on best practices, regulations and individual experience.

None of the four algorithms significantly outperformed the others, despite some of them being particularly suited to this kind of situation from a theoretical point of view. More research is needed to determine why this was the case. It is fairly likely that there is some further unexplored regularity in the behaviour of the formation.

The use of linear regression introduced certain limitations to the model. While linear regression may be sufficient for situations of low cross-entropy (e.g. where the formation is sparse), it may not deliver good predictions for dense formations. Further research can be conducted to see whether artificial intelligence methods may deliver better results, considering the premise of the universal approximator^49^.

Simulations were extensively used in this research. Although the simulator closely reflects the actual behaviour of a drone, it currently only allows for 2D simulations of rotorcraft drones. Further work is needed to include 3D simulations so that these results can be generalised to other formations and other types of drone.

Our current research uses the outcome of this work as a baseline. Predictive models that use artificial intelligence to achieve better predictability are under investigation, and have yielded promising results. We are also working with more complex cases, such as where two formations interfere, or where the intruder is able to execute its own collision avoidance algorithm. We expect to eventually develop a comprehensive model of disturbances in swarm formations of drones.

## Data Availability

The dataset generated and analysed during the current study are available in the Kaggle repository: https://www.kaggle.com/datasets/martapbs/drone-swarm-entropy.
